# Patterns of objectively measured sedentary time in 10- to 12-year-old Belgian children: an observational study within the ENERGY-project

**DOI:** 10.1186/s12887-017-0894-9

**Published:** 2017-06-14

**Authors:** Maïté Verloigne, Nicola D. Ridgers, Mai Chinapaw, Teatske M. Altenburg, Elling Bere, Wendy Van Lippevelde, Greet Cardon, Johannes Brug, Ilse De Bourdeaudhuij

**Affiliations:** 1 Department of Movement and Sport Sciences, Ghent University, Watersportlaan 2, 9000 Ghent, Belgium; 2 Research Foundation Flanders (FWO), Egmontstraat 5, 1000 Brussel, Belgium; 3 Deakin University, Geelong, Australia, Institute for Physical Activity and Nutrition (IPAN), School of Exercise and Nutrition Sciences, Burwood, Australia; 4 Department of Public and Occupational Health and the EMGO Institute for Health & Care Research, VU Medical Center, Amsterdam, the Netherlands; 5 Department of Public Health, Sport and Nutrition, University of Agder, Kristiansand, Norway; 6 Department of Public Health, Ghent University, Ghent, Belgium; 7 Department of Epidemiology and Biostatistics and the EMGO Institute for Health & Care Research, VU University Medical Center, Amsterdam, the Netherlands

**Keywords:** Sedentary time, Sedentary bouts, Children, Accelerometer

## Abstract

**Background:**

This study examined the frequency of and differences in sedentary bouts of different durations and the total time spent in sedentary bouts on a weekday, a weekend day, during school hours, during after-school hours and in the evening period in a sample of 10- to 12-year-old Belgian children.

**Methods:**

Accelerometer data were collected as part of the ENERGY-project in Belgium (*n* = 577, 10.9 ± 0.7 years, 53% girls) in 2011. Differences in total sedentary time, sedentary bouts of 2–5, 5–10, 10–20, 20–30 and ≥30 min and total time accumulated in those bouts were examined on a weekday, a weekend day, during school hours, during after-school hours and in the evening period, using multilevel analyses in MLwiN 2.22.

**Results:**

More than 60% of the participants’ waking time was spent sedentary. Children typically engaged in short sedentary bouts of 2–5 and 5–10 min, which contributed almost 50% towards their total daily sedentary time. Although the differences were very small, children engaged in significantly fewer sedentary bouts of nearly all durations during after-school hours compared to during school hours and in the evening period. Children also engaged in significantly fewer sedentary bouts of 5–10, 10–20, and 20–30 min per hour on a weekend day than on a weekday.

**Conclusions:**

Although primary school children spend more than 60% of their waking time sedentary, they generally engaged in short sedentary bouts. Children’s sedentary bouts were slightly longer on weekdays, particularly during school hours and in the evening period, although the differences were very small. These results suggest that in this age group, interventions focusing on reducing total sedentary time rather than interrupting prolonged sedentary time are needed.

**Electronic supplementary material:**

The online version of this article (doi:10.1186/s12887-017-0894-9) contains supplementary material, which is available to authorized users.

## Background

There is evidence that both the total amount of time spent in sedentary activities (i.e. volume) as well as the way sedentary time is accumulated (i.e. patterns) are associated with several health outcomes in adults, including type 2 diabetes, cardiovascular diseases, metabolic risk factors, and all-cause mortality [1–8]. In children and adolescents, the evidence is less consistent [9, 10]. A recent review of reviews reported that there was evidence of an association between screen time and obesity, blood pressure, total cholesterol, self-esteem, social behavior problems, physical fitness, and academic achievement [1]. However, it is not clear whether this association is due to sedentariness or other characteristics of screen time activities such as increased snacking during the activities [11].

Regarding the association between sedentary time accumulation and health outcomes in children, research is in its infancy and the evidence is currently limited [12–16]. Some observational studies have reported significant associations between prolonged sitting and adverse health outcomes in 10- to 14-year-old children [17]. Similar findings have been reported in specific subgroups such as boys [18], children with low physical activity levels [19] or children with a family history of obesity [20]. More recently, a study of 7- to 10-year-old children found a significant positive association between sedentary bouts of 5–10 min and cardiometabolic biomarkers [21]. However, experimental studies that have examined acute effects of sedentary time have reported mixed findings. Belcher and colleagues demonstrated that interrupting sedentary time every 20 minutes with 3-min moderate intensity walking improved metabolic function in 7- to 11-year-old children [22]. In contrast, Saunders and colleagues found that interrupting prolonged sitting every 20 min with 2 min of light intensity walking was not related to changes in cardio-metabolic markers in 10- to 14-year-old children [23]. Given the high prevalence of sedentary time in children [24] and the ever increasing choices of new digital media, it may be necessary to intervene early in life. Moreover, setting up healthy behaviours early in life might lead to established habits in adulthood. The development of interventions should be guided by objective, reliable, and valid information about children’s sedentary patterns (i.e. how is sedentary time accumulated) [25]. In recent years, guidelines such as the Australian sedentary behaviour guidelines for children and adolescents have included recommendations that specifically focus on breaking up long periods of sitting as often as possible [26]. However, it is currently not known what constitutes a long period of sitting time in children. It is clear that we need to increase our understanding of children’s sedentary patterns.

In the current study, we define sedentary patterns as how (i.e. how much time children spend in bouts of different duration and the frequency of these bouts) and when children accumulate their sedentary time (i.e. specific time periods). Currently, little research has been conducted to examine how and when children accumulate their sedentary time. Two studies have described patterns of objectively measured sedentary time in adolescent females. Harrington and colleagues [25] investigated the number of sedentary bouts of different durations that ranged from less than 1 min to more than 40 min on week- and weekend days, as well as during school hours and during after-school hours, in 111 15- to 18-year-old girls using activPAL inclinometers. Each epoch that had 15 s of uninterrupted sitting or lying was classified as the beginning of a sedentary bout, which continued until the next 15-s epoch of standing or stepping was identified. Their study indicated that the girls engaged in a greater number of sedentary bouts of more than 20 min on weekdays than on weekend days and during school hours than during after-school hours. Carson and colleagues [27] also longitudinally examined sedentary bouts lasting at least 10, 20 and 30 min on week- and weekend days and during school hours, during after-school hours and in the evening period among 655 adolescent girls using accelerometers. They defined a bout as a continuous period of sedentary time that stopped when the counts for a 30-s epoch exceeded the sedentary time cut-point of 100 counts per minute (cpm). All sedentary bout durations were more prevalent on weekdays than on weekend days, and in the evening period compared to the school and the after-school period. There was also an increase of at least 30% for all bout lengths between baseline (mean age 13.5 years) and follow-up (mean age 14.9 years).

In addition to these studies conducted in adolescents, two studies have objectively assessed sedentary bouts in children. Abbott and colleagues [28] found that 10- to 12-year-old children spent more time in prolonged sedentary time and had fewer breaks during school hours when compared to non-school hours, although only sedentary bouts of at least 30 min were examined. The authors defined a sedentary bout as an uninterrupted, 30 min or more sequence of sedentary time, using the 100 cpm cut-point. A break was defined as an interruption in sedentary time of any duration with an accelerometer count larger than 100 cpm. In contrast, Altenburg and colleagues [29] found that 10- to 13-year-old children spent most of their sedentary time in bouts that lasted more than 5 min in duration, whereas bouts of more than 20 min were rare. Using a 15-s epoch, sedentary bouts were defined as periods of consecutive minutes below the sedentary time cut-point of 100 cpm. However, the way in which children accumulated their sedentary time during specific periods of the day was not investigated in that study. Such information will be important for identifying intervention strategies to break up prolonged sitting throughout the day as well as during specific time periods of the day.

Therefore, the purpose of this study was to examine the frequency of and differences in sedentary bouts of different durations (bouts of 2–5, 5–10, 10–20, 20–30, and ≥30 min) and the total time spent in sedentary bouts on a weekday, weekend day, during school hours, during after-school hours, and in the evening period in a sample of 10- to 12-year-old Belgian children. Based on previous findings [25, 27–29], it was hypothesised that children would engage in longer sedentary bouts on a weekday compared to a weekend day, and during school hours (prolonged sedentary time during lessons) and in the evening period (which provides opportunities for discretionary screen time) compared to during after-school hours.

## Methods

### Procedure

For the purpose of this study, accelerometer data collected from Belgian children participating in the intervention study as part of the ENERGY project were analysed [30, 31]. A convenience sample of twelve primary schools were selected from Flanders, Belgium, of which ten agreed to participate. The schools were located in East- and West-Flanders. All schools were public schools, with the total number of pupils enrolled ranging from 100 to 544. Schools were asked to report the percentage of children coming from (or living in) families with few economic resources (i.e. low income families, families who receive transfer incomes). This percentage is collected yearly by the Flemish government to define the pupil characteristics per school. Based on this information, schools are provided with additional financial support in order to provide equal opportunities in education. Two schools reported that less than 5% of children came from families with few economic resources, two schools reported a percentage between 5 and 9%, five schools between 10 and 19%, and one school between 20 and 29%. All children in Grades 5 and 6 were invited to participate in the study (*n* = 772, with the majority of pupils having been born in 1999 and 2000). School visits occurred on weekdays in September–October 2011. Consenting parents and their children were asked to complete a questionnaire that assessed demographic factors (including child’s sex and age), specific sedentary behaviours such as television watching and computer use and determinants of sedentary behaviours. These questionnaire data were not used for the present paper. All children were also asked to wear an accelerometer for 1 week to collect objective sedentary time and patterns data. The study is registered in the International Standard Randomized Controlled Trial Number Register (registration number: ISRCTN34562078). The Belgian study protocol was approved by the ethical committee of the Ghent University Hospital (B67020097212).

### Measurement of sedentary pattern variables

#### Instrumentation

Accelerometers were used to measure sedentary bouts of different durations, and the total time accumulated in those sedentary bouts. Since all data were collected in a short time frame (6 weeks), four models of ActiGraph accelerometers (Pensacola, FL) were used, namely the uniaxial GT1M (3.8 cm × 3.7 cm × 1.8 cm; 27 g), and triaxial GT3X (3.8 cm × 3.7 cm × 1.8 cm, 27 g), GT3X+ (4.6 cm × 3.3 cm × 1.5 cm, 19 g), and ActiTrainer (dimensions: 8.6 cm × 3.3 cm × 1.5 cm, 51 g). The accelerometers were randomly allocated to participating children who were asked to wear it on their right hip, which was secured by an elastic belt. However, since there were significant differences in several of the outcome variables between the ActiTrainer and the other models, it was decided to remove data from children who wore an ActiTrainer from the analyses. Only the vertical axis output of the other accelerometer models was used for the present study, since there was one uniaxial model (i.e. the GT1M). Several studies have compared the vertical axis output of two or more of the models used in the present study. Robusto and Trost [32] found that there was strong agreement between data collected using the GT1M, GT3X, and GT3X+ in children and adolescents, whilst another study confirmed that the GT1M and GT3X+ outputs were comparable for 9-year-old children [33]. Finally, the vertical axis output has been shown to be comparable between the newer models (GT1M, GT3X, GT3X+) [34]. As such, it is acceptable for researchers and practitioners to use the vertical axis output from a combination of newer models within a given study [32–34]. The ActiGraph accelerometers have acceptable reliability and validity for use in a child population [35].

#### Measurement protocol

Children were asked to wear the accelerometer for seven consecutive days, including two weekend days. They were instructed to wear the accelerometer during all waking hours, except during water-based activities. Accelerometer data were initially downloaded using ActiLife v.5.7.4 software (ActiGraph, Pensacola, FL) and were then processed using a customized Excel macro. A 15-s epoch measurement interval was selected [36, 37]. Non-wear time was calculated as periods of more than 60 min of consecutive zero counts [15]. To compare between time periods within a weekday, children who provided at least 2 weekdays of a minimum of 10 h wear time were included. To compare between week- and weekend days, children who provided at least 2 weekdays with a minimum of 10 h wear time per day and 1 weekend day with a minimum of 8 h wear time were included [38]. The cut-point for sedentary time was set at 100 cpm, which has been found to provide a good estimate of sedentary time in children [39, 40]. As the focus of the present paper was on sedentary time and patterns, the amount of time children spent in light, moderate and vigorous physical activity is not described in the paper. We also calculated the total sedentary time per specific time period, which was expressed as a percentage of the total wear time.

For the purpose of this study, the following sedentary bout durations were calculated: (a) 2–5 min (which can be considered very short bouts), (b) 5–10 min, (c) 10–20 min, (d) 20–30 min, and (e) ≥30 min. For each bout length, a sedentary bout was defined as a continuous period of sedentary time for the equivalent amount of minutes (with zero tolerance time allowed). The sedentary bout stopped when the counts for a 15-s epoch exceeded the sedentary time cut-point (i.e. 25 counts per 15 s) [10, 29]. No tolerance was allowed in the sedentary bouts, as previous studies shown that interruptions can attenuate the inverse health effects of prolonged sedentary time in adults [41, 42]. In addition, Altenburg and colleagues [29] investigated the association between various definitions of sedentary bouts (i.e. allowing 0, 30 or 60 s exceeding 100 cpm) and health indicators in children, finding that a greater number of associations became significant when no tolerance was allowed within sedentary bouts.

In this paper, we report the absolute number of sedentary bouts per specific time period, as well as the number of bouts per hour in order to facilitate comparisons between time periods whilst taking wear time into account. Additionally, the total amount of time accumulated in sedentary bouts was calculated for each bout length. We report the absolute time accumulated in sedentary bouts (expressed in minutes) and the proportion of the total sedentary time of the specific time period (expressed as a percentage) in order to take wear time into account. All outcomes were calculated separately for week- and weekend days. For weekdays, three specific time periods were examined: (a) during school hours (8:30 a.m. - 4:00 p.m.), (b) after-school hours (4:00–6:00 p.m.), and (c) in the evening period (6:00–10:00 p.m.) [43]. The included schools started between 8:20 and 8:35 a.m. and ended between 3:35 and 4:15 p.m. However, as the end time sometimes differed between school days within one school, one start time (8:30 a.m.) and one end time (4:00 p.m.) was used to determine the school hours’ time period. The use of 6:00 p.m. as the cut-off for the after-school hours period versus the evening period has been used for standardising studies that examine behaviour during specific time periods of the weekday [44]. The dataset is included as Additional file 1.

### Statistical analyses

MLwiN 2.22 (Centre for Multilevel Modelling, University of Bristol, UK) was used to describe sample characteristics, to calculate mean values of the different outcome variables, and to perform the statistical analyses. For all bout durations, we investigated whether there were any significant differences in the number of sedentary bouts per hour and the total time accumulated in those bouts (expressed as a percentage) per specific time period (during school hours versus during the after-school hours and the evening period; weekday versus weekend day). Therefore, a multilevel repeated measures analysis was performed. Multilevel modelling (two-level: pupil, school) was used to account for the clustering of children in schools. The outcome variables were initially checked for normality and the skewed variables (bouts/h and time accumulated in bouts of 20–30 and ≥30 min) were log-transformed for analyses. For ease of interpretation, the non-transformed mean values are reported in the tables. The children’s age and sex were included as covariates. Statistical significance was set at *p* < 0.05.

## Results

### Sample characteristics

In total, 740 of the 772 children attending the ten schools provided informed consent from their parents to participate in the study (96%). Of those children, 643 children provided valid accelerometer data on two weekdays (90%) and 565 children provided valid accelerometer data on two weekdays and one weekend day (76%). After removing the children who wore an ActiTrainer accelerometer from the analyses, 577 children had valid data on two weekdays and 502 children on two weekdays and one weekend day. There were no differences regarding the children’s age or sex between these two groups. Attrition analyses that compared children with valid data on weekdays and/or weekend days with children who did not have any valid data showed no significant difference for age, but boys were more likely to have invalid data compared to girls. The mean age of the sample with valid accelerometer data was 10.9 (SD 0.7) years old (53% girls). With regard to the different ActiGraph accelerometer models, 46.5% of the sample wore a GT1M, 26.6% wore a GT3X, and 26.9% wore a GT3X+. The mean wear time was 829.3 (SD 73.5) minutes on weekdays, and 746.0 (SD 123.3) minutes on weekend days.

### Sedentary bouts

Table 1 shows the mean values of the total number of sedentary bouts and the bouts per hour for each bout length per specific time period. Children predominantly engaged in sedentary bouts lasting 2–5 min, with an average of 45 bouts on a weekday (3.33 per hour) and 40 bouts on a weekend day (3.28 per hour). The average number of sedentary bouts per hour of ≥10 min was less than 1 on weekdays, weekend days, and during all specific time periods. Although the differences were very small, children engaged in significantly fewer sedentary bouts of 5–10, 10–20, 20–30 and ≥30 min per hour during after-school hours compared to during school hours and in the evening period, and fewer sedentary bouts of 5–10 and ≥30 min per hour during school hours than in the evening period. Children engaged in significantly fewer sedentary bouts of 5–10, 10–20, and 20–30 min on a weekend day (1.05, 0.37, 0.08 bouts per hour, respectively) than on a weekday (1.13, 0.43, 0.09 bouts per hour, respectively).

**Table 1:** Sedentary bouts of different lengths for specific time periods

	During school hours weekday (8:30 a.m. - 4:00 p.m.)	After-school hours weekday (4:00-6:00 p.m.)	Evening period weekday (6:00-10:00 p.m.)	Weekday	Weekend day
SED bouts 2-5 min					
Total bouts	25.06	6.97	10.38	45.46	40.22
Bouts/h	3.40	3.35	3.41	3.33	3.28
SED bouts 5-10 min					
Total bouts	8.57	2.11	3.74	15.68	12.99
Bouts/h	1.16^b,c^	1.06^a,c^	1.26^a,b^	1.13^d^	1.05
SED bouts 10-20 min					
Total bouts	3.30	0.55	1.29	6.01	4.69
Bouts/h	0.45^b^	0.32^a,c^	0.47^b^	0.43^d^	0.37
SED bouts 20-30 min					
Total bouts	0.67	0.07	0.23	1.25	0.95
Bouts/h	0.09^b^	0.05^a,c^	0.09^b^	0.09^d^	0.08
SED bouts ≥30 min					
Total bouts	0.29	0.02	0.14	0.82	0.67
Bouts/h	0.04^b,c^	0.03^a,c^	0.06^a,b^	0.06	0.05

*SED* sedentary

^a^significantly different from during school hours period

^b^significantly different from after school hours period

^c^significantly different from the evening time period

^d^significantly different from a weekend day

### Time accumulated in sedentary bouts

**Fig. 1:**
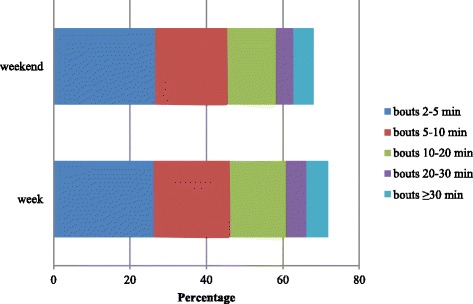
Contribution of sedentary bouts of different lengths to total sedentary time during school hours, after-school hours and in the evening period (expressed in percentage)

**Fig. 2:**
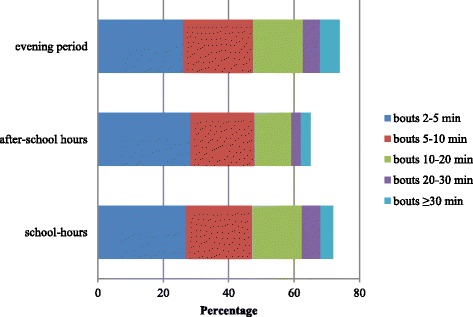
Contribution of sedentary bouts of different lengths to total sedentary time on a week- and weekend day (expressed in percentage)

Table 2 shows the mean total time accumulated in sedentary bouts for each bout length and the accumulated time as a proportion of the total sedentary time. Despite the differences being very small, children accumulated significantly less time in sedentary bouts lasting 10–20 min (11.3%) as a proportion of their total daily sedentary time during after-school-hours than during school hours (15.3%) and in the evening period (15.2%). Similar findings were also found for bouts of 20–30 min (2.9%) as a proportion of their total daily sedentary time during after-school hours when compared to during school hours (5.6%) and in the evening period (5.2%). Children also accumulated significantly less time in sedentary bouts of 5–10 and ≥30 min as a proportion of the total sedentary time during school hours (20.3%, 3.8%) and during after-school hours (19.6%, 2.9%) than in the evening period (21.3%, 6.1%). Finally, children accumulated significantly less time in sedentary bouts of 5–10 min (18.8 versus 19.9%), 10–20 min (12.8 versus 14.6%), and 20–30 min (4.5 versus 5.4%) as a proportion of their total sedentary time on a weekend day than on a weekday. Figures 1 and 2 visually show the contribution of each bout duration to the total sedentary time (expressed as a percentage). The sum of the different percentages does not add up to 100%, which means that the remaining proportion of the total sedentary time was spent in sedentary bouts lasting less than 2 min in length.

**Table 2:** Total time accumulated in sedentary bouts of different length for specific time periods

	During school hours weekday (8:30 a.m. - 4:00 p.m.)	After-school hours weekday (4:00-6:00 p.m.)	Evening period weekday (6:00-10:00 p.m.)	Weekday	Weekend day
SED bouts 2-5 min					
Total time accumulated (min)	76.1	20.8	31.4	137.9	121.3
Proportion of SED time (%)	26.9^b,c^	28.3^a,c^	26.2^a,b^	26.3	26.7
SED bouts 5-10 min					
Total time accumulated (min)	58.4	14.2	25.5	107.0	88.7
Proportion of SED time (%)	20.3^c^	19.6^c^	21.3^a,b^	19.9^d^	18.8
SED bouts 10-20 min					
Total time accumulated (min)	44.5	7.3	17.2	81.0	63.2
Proportion of SED time (%)	15.3^b^	11.3^a,c^	15.2^b^	14.6^d^	12.8
SED bouts 20-30 min					
Total time accumulated (min)	16.1	1.6	5.5	29.9	22.7
Proportion of SED time (%)	5.6^b^	2.9^a,c^	5.2^b^	5.4^d^	4.5
SED bouts ≥30 min					
Total time accumulated (min)	11.2	0.7	5.7	33.8	28.9
Proportion of SED time (%)	3.8^c^	2.9^c^	6.1^a,b^	5.7	5.3
SED time					
Total SED time (min)	284.1	71.5	117.3	532.8	463.9
Proportion of wear time (%)	64.0^b,c^	59.8^a,c^	65.9^a,b^	64.1^d^	61.9

*SED* sedentary

^a^significantly different from during school hours period

^b^significantly different from after school hours period

^c^significantly different from the evening time period

^d^significantly different from a weekend day

### Total sedentary time

Table 2 shows the mean total sedentary time per time period, expressed in minutes, and as a proportion of the accelerometer wear time. Children spent significantly less time being sedentary during after-school hours (59.8%) than during school hours (64.0%) and in the evening period (65.9%). They were also significantly less sedentary during school hours than in the evening period. Finally, children engaged in significantly less sedentary time on a weekend day (61.9%) than on a weekday (64.1%).

## Discussion

This study found that 10- to 12-year-old children typically accumulate their sedentary time in very short bouts. Sedentary bouts lasting more than 20 min were not common in this age group. These results support the findings of Altenburg et al. [29] who found that 10- to 13-year-old children engage, on average, in only three bouts of more than 20 min per day. This indicates that primary school children generally do not engage in prolonged sedentary time. In addition, sedentary bouts of 2–5 and 5–10 min contributed almost 50% towards children’s total daily sedentary time. Approximately 15 and 10% of the children’s total sedentary time was spent in bouts of 10–20 min and more than 20 min, respectively. Interestingly, these results imply that more than 25% of children’s sedentary time is accumulated in bouts that do not exceed 2 min, as the sum of the above mentioned percentages does not add up to 100%. Consequently, whilst children’s total sedentary time during the day and in specific time periods was high (up to 65% of a period), they rarely sit down for a prolonged period of time. This is in contrast to research conducted in adults that has found that they spend about 37% of their total sedentary time in sedentary bouts that last at least 20 min on non-work days and almost 50% on work days [45].

Some differences between week- and weekend days were observed. Children engaged in slightly more sedentary bouts lasting 5–10, 10–20, and 20–30 min on weekdays. This is in line with previous studies conducted with adolescent females where bouts of several durations were more prevalent on weekdays than on weekend days [25, 27]. On weekdays, children engaged in somewhat longer bouts at school and during the evening period, whilst more time was accumulated in bouts of 5–10 min and more than 30 min in the evening period compared to the school period. This is line with the findings of previous studies conducted in adolescent females. Harrington and colleagues [25] also found that a greater number of sedentary bouts occurred during school hours than during after-school hours, whilst Carson and colleagues [27] confirmed that a greater number of sedentary bouts of all durations occurred in the evening period compared to during school hours and during after-school hours. Abbott and colleagues [28] also found that children engaged in more sedentary bouts during school hours compared to non-school hours, although only bouts of more than 30 min were investigated. Despite the fact that our study shows that longer bouts occur at school and in the evening period, it must be acknowledged that children did not engage in prolonged sitting in those specific periods and that the differences between time periods were very small. Nevertheless, the total amount of time spent sedentary was 64% during weekdays and 62% during weekend days. For weekdays, children spent 64% and more than 65%, respectively, of their time at school and in the evening period being sedentary. This was significantly higher than during the after-school hours period where ‘only’ 59.8% of their time was spent sedentary. This percentage is comparable to previous studies that have examined children’s levels of sedentary time per day or during specific time periods [46–49].

Overall, our results suggest that the total volume of sedentary time that children engage in seems to be more of a problem in this age group compared to how this sedentary time is accumulated, especially on weekdays, during school hours and in the evening period. The high prevalence of total sedentary time in combination with a high frequency of interruptions might explain the inconsistent findings with regard to the health consequences of sedentary behaviour in youth. Moreover, it might also explain why interventions focusing on interrupting children’s sedentary time have had limited effects to date [43]. As such, it could be recommended that interventions focus on decreasing the total time amount of time that children spend sedentary, although more research is needed to determine how much sedentary time is harmful to children’s health both in the short- and long-term [10]. A possible strategy to reduce total sedentary time is through the use of standing desks in primary schools [50]. Another strategy could be to let children perform specific tasks while standing, which is of course less drastic and less expensive for a school to implement. However, we acknowledge that to date, the evidence that standing reduces the potential adverse health effects of excessive sedentary time is inconsistent and has been predominantly investigated in adults [51–53]. For the evening hours, a possible intervention strategy to reduce sedentary time could be to use a monitoring device attached to the television or computer as a cue to limit these sedentary behaviours [54], although we have to keep in mind that children use multiple devices for entertainment (e.g. smart phones, tablets). However, we should be cautious about proposing such intervention strategies, since a systematic review that investigated the effectiveness of intervention strategies focusing exclusively on reducing sedentary time in children found no convincing evidence for any of these approaches. The authors recommended that mediation analyses are needed in the future in order to identify the most effective strategies [55].

An important strength of the current study was the objective measurement of sedentary time patterns in a relatively large sample. Although accelerometers are unable to capture posture, using the cut-point of 100 cpm has been shown to accurately measure actual sitting time, as opposed to higher cut-points that are more likely to capture both sitting and standing time [40]. Another strength was the calculation of different bout durations, ranging from 2 to 5 min to ≥30 min, in order to provide detailed information on how sedentary time is accumulated in this age group. However, it must be acknowledged that analysing delimited bouts in separate analyses would not be relevant when analysing the relationship with health outcomes, since only a part of sedentary time is calculated [14]. There are also some limitations that need to be acknowledged. First, there is currently no consensus on how to define sedentary sedentary bouts, and breaks in sedentary time [14, 15]. For example, some studies (including this study) examine activity accumulation in terms of set bout ranges (i.e. the delimited approach) [19, 20], some examine sedentary bouts of at least a certain duration [17, 29], and others investigate ‘usual’ bout duration [56, 57]. These differences make it hard to compare studies. Another potential limitation is that we averaged the week- and weekend days for children. A study conducted with 9- to 11-year-old children showed that the total sedentary time accumulated differed according to a specific day [58]. It is also possible that there is day-to-day variability with regard to sedentary patterns, so future research should verify this. Another limitation is the use of different ActiGraph accelerometer models due to the limited time period to measure the children, despite the fact that it is acceptable to use different models together in one study [32]. A final limitation is the convenience sampling strategy, which might affect the generalisability of the results, since most schools had a low percentage of pupils coming from families with low economic resources. The statistics for Flanders show that the average percentage of children is between 18.6–21.4% [59].

## Conclusion

In order to inform future interventions, it is important to understand how and when specific population groups accumulate their sedentary time. Belgian 10- to 12-year-old children engage in high levels of sedentary time (more than 60% of their waking time), particularly during school hours and in the evening period on weekdays. However, these children do not engage in many sedentary bouts of longer durations, and only small, albeit significant, differences in bout durations were found across different periods of children’s waking hours. Thus, interventions focusing on reducing the total volume of sedentary time seem more appropriate than focusing on reducing sedentary bout durations in this age group.

## Additional file

Additional file 1: Dataset. Sedentary patterns of 10- to 12-year old children. (XLS 1444 kb)

## Abbreviations

Cpm: Counts per minute
SD: Standard deviation
